# Complete Blood Count and Biochemistry Reference Intervals for Healthy Adult Donkeys in the United States

**DOI:** 10.3390/ani14142018

**Published:** 2024-07-09

**Authors:** Erin L. Goodrich, Julie L. Webb

**Affiliations:** Department of Population Medicine and Diagnostic Sciences, Cornell College of Veterinary Medicine, Ithaca, NY 14850, USA; jlw444@cornell.edu

**Keywords:** donkey, equid, hematology, serum biochemistry, clinical pathology

## Abstract

**Simple Summary:**

Although donkeys and horses share many similarities, their clinical presentations of disease and other physiological attributes can be quite different. Veterinarians working on donkeys can find it quite challenging to definitively diagnose their disease condition based on physical examination findings alone and must frequently rely on clinical pathology data from bloodwork. This study aimed to utilize healthy adult captive and free-roaming donkeys from across the United States to create robust reference intervals for complete blood count and serum biochemistry values. The findings from this study will help veterinarians with the interpretation of these common clinical pathology diagnostic tests in this unique equid species.

**Abstract:**

Previous hematologic and serum biochemistry reference interval (RI) values have been established for donkeys in various geographic regions, life-stages, or for specific donkey breeds. The last extensive investigation establishing RIs for adult donkeys in the United States (U.S.) was published over three decades ago. We aimed to establish updated robust RIs using a reference population of apparently healthy adult donkeys from across the U.S. Standard sized (*n* = 102), miniature (*n* = 17), and mammoth (*n* = 1) donkeys from four different states were enrolled, with 20% of the study population including donkeys captured directly from the wild in Death Valley National Park, CA. RIs were established in accordance with the American Society for Veterinary Clinical Pathology and Clinical Laboratory Standards Institute guidelines. The findings will assist practitioners with the interpretation of their complete blood count and biochemistry panel results in U.S. donkeys. This study also highlights a comparison of results for some important analytes in U.S. donkeys compared to U.S. horses and previously established donkey RIs.

## 1. Introduction

Donkeys are a unique equine species with many behavioral traits that they have retained from their ancestors, the African Wild Asses [1]. Some of these unique traits include their subtle display of disease. Donkeys often demonstrate only vague, elusive signs of illness or pain, such as dull demeanor, increased frequency or time spent lying down, decreased appetite, or isolation from their herd-mates [2,3,4]. The changes in behavior that an astute owner, caretaker, or veterinarian may observe might not even obviously correlate to the body system that is affected or the stage of disease. For instance, painful hoof disorders in mammoth asses may be more likely to result in recumbency than painful gastrointestinal disorders [4]. Veterinarians must perform thorough physical examinations of donkey patients but often need to also rely heavily on coupling those findings with supporting laboratory data to make an accurate diagnosis.

Reference interval (RI) information specific to donkey clinicopathologic data is limited. Furthermore, most of the current donkey RI literature utilizes breed, geographic location, or life-stage-specific populations that may not be applicable to larger and more varied populations [5,6,7,8,9,10,11,12]. One of the most extensive donkey RI studies to date involved 138 samples from donkeys living in a sanctuary in the United Kingdom (UK) [13]. In that study, Burden and colleagues compared their findings to horses and to a previous UK donkey RI study, finding only limited transferability between the various populations. This important study highlights the need for RIs to represent the appropriate species and population and for the intermittent reassessment of reference intervals, as changes in management and other influences may affect intervals over time.

The New York State Animal Health Diagnostic Center (AHDC) receives approximately 500 hematologic or serum biochemical test requests on donkey blood samples annually. To the authors’ knowledge, the last extensive RI study involving donkeys (*n* = 217) in the continental U.S. was published in 1990 [14]. Our goal in this study was to utilize the guidance of the American Society of Veterinary Clinical Pathology (ASVCP) to establish hematologic and serum biochemistry reference intervals at the AHDC for adult donkeys. We hypothesized that our reference intervals would differ from those previously established for donkeys in other publications and those previously established for horses by the AHDC. The authors sought to assess hematologic and biochemical RIs specific to US donkeys to assist practitioners who rely heavily on these objective diagnostic tests to accurately categorize disease states in this stoic species.

## 2. Materials and Methods

### 2.1. Ethics Approval

This study, under the umbrella protocol “Laboratory test reference intervals, method validation and testing for diagnostic utility of laboratory assays”, was performed under an approved International Animal Care and Use Committee (IACUC) protocol. Signed owner consent was obtained for all sample collection.

### 2.2. Animals and Sample Collection

First, 120 donkeys were sampled between February 2020 and June 2021 from several locations across the U.S., representing geographic locations typical of submissions to the AHDC. Samples were collected as part of routine/annual exams, herd health visits, and castration procedures. Inclusion criteria were apparently healthy donkeys (as assessed by the veterinarian obtaining the samples) greater than or equal to two years of age, with no known health problems diagnosed in the past six months. Donkeys must not have received any pharmaceutical products other than anthelminthics in the past six months, and must have been determined to have a body condition score (BSC) of 2, 3, or 4 on a 5-point scale, by the submitting veterinarian at the time of sample collection [15]. No specific requirements were made regarding diet or time of last meal. When available, the actual age of the donkey was obtained from birthdates recorded in health records; otherwise, it was estimated by the veterinarian, based on dentition. Donkeys were also characterized based on their size as miniature (less than 36 inches at the withers), mammoth (greater than or equal to 56 inches at the withers), or standard (measuring between those sizes at the withers) [16]. The sex of the donkey was also recorded (intact male, castrated male, or female).

Venipuncture was performed on each donkey from the left or right jugular. Whole blood was collected into either a 7 mL Covidien Monoject™ Vacutainer K3-EDTA tube (Medtronic, Dublin, Ireland) or a 4 mL BD Vacutainer K2-EDTA tube and a BD Vacutainer^®^ serum tube (Becton Dickinson, Franklin Lakes, NJ, USA). Within one hour of collection, two unstained, air-dried, blood smears were made using the EDTA whole blood sample. For most samples, the serum tubes were centrifuged within four hours of collection, and serum was removed from the blood clot and transferred to another BD Vacutainer^®^ serum tube. A few veterinarians were unable to centrifuge samples themselves, so 10 samples were submitted as clotted whole blood in the serum tube, and centrifugation was performed at the AHDC. The blood and serum samples were kept chilled on ice packs or refrigerated until overnight shipment on ice packs to the AHDC within 48 h of sample collection. The blood smear slides were placed in slide mailers and protected from contact with ice packs during shipment.

### 2.3. Laboratory Analyses

All samples were analyzed within 24 h of receipt. EDTA whole blood was utilized for all hematology testing, and serum was utilized for all biochemistry testing. The total protein analyte of the complete blood count (CBC) was determined by refractometry. Hematocrit, hemoglobin concentration (Hb), red blood cell count (RBC), mean corpuscular volume (MCV), mean corpuscular hemoglobin (MCH), mean corpuscular hemoglobin concentration (MCHC), red cell distribution width (RDW), platelet count, mean platelet volume (MPV), and white blood cell count (WBC) were determined using a Siemens Advia 2120i automated hematology analyzer (Siemens Medical Solutions, Malvern, PA, USA). The differential WBC count was derived by manual count of 100 cells in Wright-Giemsa-stained smears. Biochemical parameters and their associated methods are listed in Table 1 and were measured using a Cobas 501 automated serum chemistry analyzer (Roche Diagnostics, Indianapolis, IN, USA). Both laboratory analyzers are evaluated daily for performance using commercially available quality-control material and calibrated as needed. At the time of testing, the laboratory participated in two external quality assurance programs.

### 2.4. Statistical Analysis

Data were analyzed using Reference Value Advisor v.2.1 (Ecole Nationale Veterinaire de Toulouse, Toulouse, France), and reference intervals were calculated according to the guidelines of the American Society for Veterinary Clinical Pathology (Madison, WI, USA) [17,18]. Tukey’s method was used to assess for outliers and suspect data. These values were retained unless there was convincing error or a result pattern inconsistent with health. The distribution of the data was evaluated by the Anderson–Darling method, with a *p*-value < 0.05 indicating non-Gaussian distribution. The symmetry of the data was evaluated using the runs test, with a *p*-value < 0.05 indicating asymmetry. The parametric method was selected for Gaussian distributions. The nonparametric method was selected when data had a non-Gaussian and asymmetrical distribution, both before and after Box–Cox transformation. The bootstrap method was used to establish confidence intervals. In any instance where outliers were removed, the data were re-analyzed using the methods above.

## 3. Results

The study population consisted of 28 owned donkeys in New York (23%), 6 in Massachusetts (5%), 18 in California (15%), 44 in Texas (37%), and 24 (20%) free-roaming donkeys upon capture from the wild in Death Valley National Park, California. Most donkeys sampled were standard-sized (*n* = 102), followed by miniature (*n* = 17), and only a single mammoth, with 55 being intact males, 16 castrated males, and 49 females.

A total of 120 EDTA whole blood samples (and blood smears) were collected for CBC analysis, but 9 samples were rejected due to clotting in the blood tube, and 2 samples were rejected because it was discovered that they were collected from foals less than six months of age. A total of 120 serum samples were collected for biochemistry analysis, but 1 was lost during shipping, and 2 samples were rejected because it was discovered that they were collected from foals less than six months of age. The majority of suspect and outlier data was retained as the population was well defined with general health established. The outliers removed are detailed in Table 2. CBC and biochemistry reference interval data are presented in Table 3 and Table 4, respectively.

## 4. Discussion

One goal in this study was to establish reference intervals for hematologic and biochemistry values in healthy adult donkeys at the AHDC, as a means of bolstering submitting veterinarians’ ability to make accurate diagnoses in this unique equid species. To best represent the donkey population that is typically tested at the AHDC, the reference population was assembled from a wide geographic range throughout the U.S. and included mostly standard-sized donkeys, followed next by miniature donkeys and a single mammoth donkey. Intact male donkeys did make up a large proportion of the CBC (49%) and biochemistry panels (46%) performed for this study because these samples were convenience samples obtained just prior to a scheduled mass castration event at the facility where the animals were housed. Wild donkeys roam freely across much of the Western U.S. The Bureau of Land Management (BLM) estimates there are currently over 14,000 free-roaming donkeys in herd management areas in Arizona (AZ), California (CA), Nevada (NV), Oregon (OR), and Utah (UT) as of 1 March 2024 [19]. One of the approaches that the BLM utilizes for population control is the periodic removal and capture of wild donkeys from these lands, with subsequent adoption into private care. To account for this in our reference population, approximately 20% of the CBC and chemistry data were obtained from free-roaming donkeys upon capture in Butte Valley, an area within Death Valley National Park, CA.

An additional goal of this study was to analyze at least 120 samples to establish robust reference intervals and utilize nonparametric statistical methods, a process that minimizes the effect of outliers and is recommended to best characterize a population [18]. Animal availability, submitter compliance, and sporadic sample issues, however, reduced the number of acceptable samples slightly below the intended threshold, and statistical methods were adjusted to best characterize the data for each analyte.

**Figure 1:**
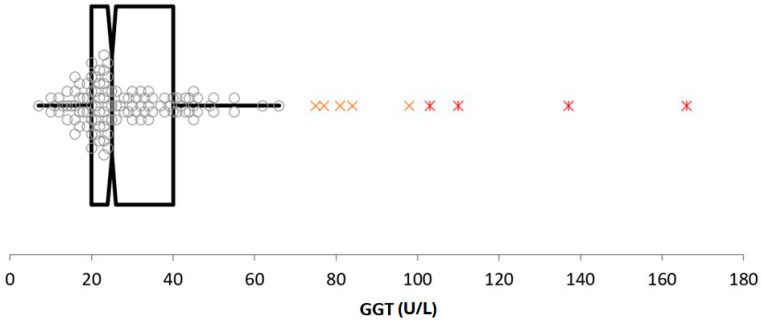
Initial GGT analysis prior to outlier removal, showing several high suspect (orange X) and outlier (red *) data points, creating a wide reference interval with a right-tail skew.

**Figure 2:**
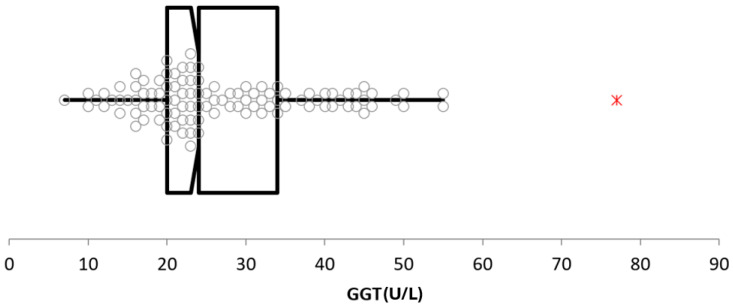
GGT analysis after outlier removal, showing a data spread and reference interval more typical of expected variation. A single outlier (red *) remains from an apparently healthy donkey.

Most outliers removed were repetitive findings deemed to be the result of common pre-analytical artifacts (e.g., uneven WBC distribution on blood smears, platelet clumping causing false thrombocytopenia, delayed serum separation from cells causing false hypoglycemia, capture and handling causing increased CK). Other outliers removed were generally a single analyte from a single specimen and had a minimal impact on the reference interval calculation. Two exceptions were GGT and lipid values. Initial GGT analysis revealed several high suspect and outlier data points, creating a wide reference interval with a right-tail skew (Figure 1). Analysis of the results revealed that 6/10 of the highest values came from a single herd whose samples were submitted as whole clotted blood sample. Previous and subsequent biochemistry bloodwork from those donkeys, sent to a different laboratory, all had significantly lower GGT levels, so an unknown artifact is presumed to have occurred in this case. Further, 3/10 of the remaining highest GGT values were then discovered to have come from a separate single herd source, and that herd was reported to have a history of liver flukes. Removal of these GGT outliers produced a data spread and reference interval more typical of the expected variation (Figure 2). A few cholesterol and triglyceride outliers were also removed from analysis. These results came from various ages and premises, but their pattern of results suggested subclinical hyperlipidemia was likely present in these individual animals. Removal of these GGT and lipid outliers was a subjective decision, as definitive disease or error could not be determined. But the authors felt that their removal was the most appropriate decision based on their combined clinical and laboratory experiences.

Reference intervals generated by this study are similar to a 2016 study by Burden and colleagues, representing 138 donkeys in the United Kingdom [13]. Of particular note is that the TG RI (0.3–2.2 mmol/L) corresponds closely to their TG RI of 0.6–2.8 mmol/L, a significant difference from an earlier large UK study [20]. Metabolic disorders in donkeys are a common problem and associated with a high morbidity and mortality rate. Hyperlipemia was previously defined as the fatty infiltration of organs with a serum TG concentration >4.4 mmol/L [21], but this study supports the theory that a lower cutoff would be more appropriate to recognize donkeys at risk for developing hyperlipemia. This study also supports the concept that species-specific reference intervals are critical to evaluate patients, as important differences were noted between the AHDC’s previously established equine (horse) RIs, previously established donkey RIs from other studies, and these newly derived donkey RIs, especially in key analytes like TGs and GGT, as shown in Table 5.

Limitations of this study included failing to meet the target of 120 samples and having some variability in sample collection and processing (e.g., some chemistry samples were not centrifuged until receipt at the AHDC). The lower number of samples likely had a minimal effect on the end results as only a few samples were excluded or lost, and guidelines are established to calculate reference intervals for smaller populations [18]. However, future studies would benefit from including extra animals and/or continuing sample collection until the target value of 120 samples is reached. Future studies may also benefit from more stringent sample collection and processing requirements, but such requirements may also limit the number of veterinarians who are able and willing to participate in sample collection.

## 5. Conclusions

This study provides updated RIs for CBC and serum chemistry analyte values in apparently healthy domestic and free-roaming adult donkeys from across the U.S. These findings will assist veterinarians with result interpretation and, thereby, aid in accurate disease diagnoses in donkeys.

## Figures and Tables

**Table 1:** Basic methodology for biochemical testing performed on the Roche Cobas.

Measurand	Method
Albumin	Bromocresol green dye-binding
AST	NADH oxidation
Electrolytes	Indirect ion-selective potentiometry
Bicarbonate	Phosphoenolpyruvate carboxylase based
Bilirubin, total	Jendrassik-Grof based- Diazo method (diazonium ion)
Bilirubin, direct	Jendrassik-Grof based- (diazotized sulfanilic acid)
Calcium	5-nitro-5′-methyl-BAPTA (NM-BAPTA)
Cholesterol	Cholesterol esterase
Creatinine kinase	Creatine phosphate cleavage
Creatinine	Modified Jaffe
GGT	L-gamma-glutamyl-3-carboxy-4- nitroanilde substrate
Glucose	Hexokinase
GLDH	Alpha-oxoglutarate substrate
Magnesium	Xylidyl blue, diazonium salt
Phosphate	Ammonium molybdate
SDH	D-fructose substrate
Triglycerides	Lipoprotein lipase
Total protein	Biuret
Urea Nitrogen	Urease- kinetic

**Table 2:** Outliers removed from analysis.

Measurand	*n*	Reason(s)
Manual WBC Differential	17	Uneven distribution of WBCs on smears causing erroneous differential percentages/counts
Platelet	21	Marked platelet clumping causing falsely decreased counts
MPV	21	Marked platelet clumping causing falsely increased MPV
TP (on CBC)	1	Moderate to marked hemolysis causing interference with refractometer
Electrolytes	1	Multiple outliers from single sample, suggesting subclinical disease
Calcium	1	Low outlier associated with low albumin, presumed artifact of decreased protein binding
Glucose	12	Very low values (<50 mg/dL) incompatible with health, presumed artifact of delayed serum separation from cells (10 of 12 outliers were received as whole clotted blood).
AST, SDH, GLDH	1	Multiple high hepatocellular enzyme outliers from a single sample, suggesting subclinical liver disease
GGT	6	Multiple high outliers associated with single herd. Samples received as whole clotted blood, suspect pre-analytical artifact
GGT	3	Multiple high outliers associated with single herd. Follow-up communication with submitter reveals history of liver flukes in herds.
Cholesterol and Triglycerides	3	Samples with both lipid values marked as suspect or outlier data (high), suggesting subclinical disease
Triglycerides	1	Single sample with marked hypertriglyceridemia, incompatible with health
CK	5	Samples with CK > 800, suggestive of traumatic handling or restraint

**Table 3:** Hematology reference intervals established from 109 donkeys using an Advia 2120 and manual differential.

Measurand	Units	*n*	Mean	SD	Median	Min	Max	*p*-Value *	Dist	Method	LRL of RI	URL of RI	CI of LRL	CI of URL
Hematocrit	L/L	109	39.1	6.5	39.0	26	54	0.184	G	P	28	53	27–29	51–56
RBC	10^12^/L	109	6.4	1.2	6.2	4.2	8.9	0.023	NG	NP	4.4	8.8	4.2–4.7	8.4–8.9
Hemoglobin	g/L	109	131	21	130	89	177	0.223	G	P	93	175	89–101	170–177
MCV	fL	109	61	3.9	61	51	70	0.162	G	P	53	68	51–54	67–69
MCHC	g/L	109	335	7	330	310	350	<0.001	NG	NP	320	350	310–320	340–350
MCH	pg	109	21	1.4	21	17	23	<0.001	NG	NP	18	23	17–18	22–23
RDW	%	109	17.3	0.9	17.1	15.9	21.6	0.753	G	P	16.1	19.4	16.0–16.2	18.9–19.9
WBC	10^9^/L	109	10.0	2.6	9.7	5.3	18.4	0.996	G	P	5.8	16.0	5.5–6.3	14.9–17.2
Neutrophil	10^9^/L	92	4.7	1.6	4.4	2.2	10.6	0.390	G	P	2.2	8.5	2.0–2.4	7.8–9.3
Lymphocyte	10^9^/L	92	4.7	2.2	4.4	1.1	11.1	0.732	G	P	1.5	9.9	1.3–1.7	8.9–10.8
Monocyte	10^9^/L	92	0.4	0.2	0.3	0	1.2	0.001	NG	NP	0	1.0	0–0.1	0.8–1.2
Eosinophil	10^9^/L	92	0.4	0.4	0.3	0	1.9	0.001	NG	NP	0	1.7	0–0	1.4–1.9
Basophil	10^9^/L	92	0	0.1	0	0	0.3	<0.001	NG	NP	0	0.1	0–0	0.1–0.3
Platelet	10^9^/L	61	197	85	195	81	396	0.003	NG	NP	84	380	81–89	322–396
MPV	fL	61	6.1	0.8	6.1	4.8	8.7	0.742	G	P	4.8	8.1	4.7–5.0	7.7–8.6
TP	g/L	108	71	5	70	56	84	0.402	G	P	60	81	59–62	79–82

Dist, Distribution; G, Gaussian; NG, non-Gaussian; NP, nonparametric; P, parametric; RI, reference interval; LRL, lower-reference limit; URL, upper-reference limit; CI, 90% confidence interval. * Anderson-Darling, <0.05 indicates non-Gaussian distribution.

**Table 4:** Biochemistry reference intervals established from 117 donkeys using a Cobas 501.

Measurand	Units	*n*	Mean	SD	Median	Min	Max	*p*-Value *	Dist	Method	LRL of RI	URL of RI	CI of LRL	CI of URL
Sodium	mmol/L	116	134	3.2	135	127	144	0.047	NG	NP	127	140	127–128	139–144
Potassium	mmol/L	116	4.6	0.6	4.5	3.1	5.8	0.545	G	P	3.4	5.7	3.3–3.6	5.6–5.8
Chloride	mmol/L	116	100	3.0	100	93	107	0.058	G	P	94	106	93–95	105–107
Bicarbonate	mmol/L	116	23	2.4	23	15	29	0.002	NG	NP	16	27	15–19	26–29
Anion Gap	mmol/L	116	17	3.2	16	11	28	0.020	NG	NP	12	25	11–12	25–28
Na/K Ratio		116	30	4.1	30	22	45	0.089	G	P	23	40	23–24	38–41
Urea nitrogen	mmol/L	117	4.3	1.5	4.3	0.7	9.3	0.001	NG	NP	1.1	7.5	0.7–1.8	6.4–9.3
Creatinine	umol/L	117	88	18	88	44	133	<0.001	NG	NP	53	133	44–53	115–133
Calcium	mmol/L	116	3.0	0.1	3.0	2.6	3.2	0.128	G	P	2.7	3.2	2.7–2.8	3.1–3.2
Phosphorus	mmol/L	117	1.2	0.2	1.2	0.7	1.8	0.741	G	P	0.7	1.6	0.7–0.8	1.6–1.7
Magnesium	mmol/L	117	0.7	0.1	0.7	0.5	1.2	0.016	NG	NP	0.5	1.0	0.5–0.6	1.0–1.2
Total protein	g/L	117	67	5	67	48	81	0.081	G	P	57	78	56–58	76–79
Albumin	g/L	117	30	4.0	30	17	37	0.015	NG	NP	20	36	17–23	35–37
Globulin	g/L	117	37	5	37	28	51	0.226	G	P	29	48	28–30	46–49
A/G Ratio		117	0.8	0.2	0.8	0.4	1.2	<0.001	NG	NP	0.5	1.1	0.4–0.5	1.1–1.2
Glucose	mmol/L	105	4.8	0.8	4.8	2.4	7.4	0.301	G	P	3.2	6.5	3.0–3.4	6.2–6.8
AST	U/L	116	367	84	364	201	667	0.744	G	P	223	555	208–238	525–587
SDH	U/L	116	4	3.2	4	2	29	<0.001	NG	NP	2	10	2–2	9–29
GLDH	U/L	116	4	2.2	3	0	14	<0.001	NG	NP	0	9	0–1	8–14
GGT	U/L	107	28	12	24	7	77	0.200	G	P	10	55	9–12	52–65
Bilirubin	umol/L	117	1.7	0.9	1.7	0	3.4	<0.001	NG	NP	0	3.4	0–0	1.7–3.4
Dir Bilirubin	umol/L	117	0	0.3	0	0	1.7	<0.001	NG	NP	0	1.7	0–0	0–1.7
Indir Bilirubin	umol/L	117	1.7	0.9	1.7	0	1.7	<0.001	NG	NP	0	1.7	0–0	1.7–1.7
CK	U/L	112	231	105	210	47	725	0.055	G	P	95	500	86–105	446–569
Cholesterol	mmol/L	114	2.0	0.4	2.0	1.3	3.2	0.281	G	P	1.4	2.4	1.3–1.5	2.8–3.1
Triglycerides	mmol/L	113	1.2	0.5	1.2	0.1	2.4	0.273	G	NP	0.3	2.2	0.2–0.4	2.0–2.3

Dist, Distribution; G, Gaussian; NG, non-Gaussian; NP, nonparametric; RI, reference interval; LRL, lower-reference limit; URL, upper-reference limit; CI, 90% confidence interval;. * Anderson-Darling, <0.05 indicates non-Gaussian distribution.

**Table 5:** A comparison of the RI for TG and GGT, highlighting differences and similarities across donkey studies and between horses and donkeys.

	TG (mmol/L)	GGT (U/L)
AHDC Donkeys (current study)	0.3–2.2	10–55
AHDC Equines *	0.2–0.7	8–33
Previous US Donkeys (Zinkl et al. 1990) [14]	NA	10–128
Previous UK Donkeys (French & Patrick 1995) [20]	0.2–4.3	8–49
Texas Donkeys (Dugat et al. 2010) [12]	0.3–1.4	NA
UK Donkeys (Burden et al. 2016) [13]	0.6–2.8	14–69

NA: Not applicable. * RIs established at the AHDC (from at least 120 adult healthy horses).

## Data Availability

The raw data supporting the conclusions of this article will be made available by the authors on request.
